# Anti-apoptotic peptide for long term cardioprotection in a mouse model of myocardial ischemia–reperfusion injury

**DOI:** 10.1038/s41598-020-75154-x

**Published:** 2020-10-22

**Authors:** Aurélie Covinhes, Laura Gallot, Christian Barrère, Anne Vincent, Catherine Sportouch, Christophe Piot, Bernard Lebleu, Joël Nargeot, Prisca Boisguérin, Stéphanie Barrère-Lemaire

**Affiliations:** 1 IGF, Université de Montpellier, CNRS, INSERM, Montpellier, France; 2 Laboratory of Excellence Ion Channel Science and Therapeutics, Valbonne, France; 3 Département de Cardiologie Interventionnelle, Clinique du Millénaire, Montpellier, France; 4 LPHI, CNRS, Université de Montpellier, Montpellier, France; 5 CRBM, CNRS, Université de Montpellier, Montpellier, France; 6 Present Address: CNRS, UMS3426, Réseau d’Histologie Expérimentale de Montpellier, BioCampus, Montpellier, France

**Keywords:** Myocardial infarction, Target validation

## Abstract

Reperfusion therapy during myocardial infarction (MI) leads to side effects called ischemia–reperfusion (IR) injury for which no treatment exists. While most studies have targeted the intrinsic apoptotic pathway to prevent IR injury with no successful clinical translation, we evidenced recently the potent cardioprotective effect of the anti-apoptotic Tat-DAXXp (TD) peptide targeting the FAS-dependent extrinsic pathway. The aim of the present study was to evaluate TD long term cardioprotective effects against IR injury in a MI mouse model. TD peptide (1 mg/kg) was administered in mice subjected to MI (TD; n = 21), 5 min prior to reperfusion, and were clinically followed-up during 6 months after surgery. Plasma cTnI concentration evaluated 24 h post-MI was 70%-decreased in TD (n = 16) versus Ctrl (n = 20) mice (p***). Strain echocardiography highlighted a 24%-increase (p****) in the ejection fraction mean value in TD-treated (n = 12) versus Ctrl mice (n = 17) during the 6 month-period. Improved cardiac performance was associated to a 54%-decrease (p**) in left ventricular fibrosis at 6 months in TD (n = 16) versus Ctrl (n = 20). In conclusion, targeting the extrinsic pathway with TD peptide at the onset of reperfusion provided long-term cardioprotection in a mouse model of myocardial IR injury by improving post-MI cardiac performance and preventing cardiac remodeling.

## Introduction

Cardiovascular diseases (CVD) are the primary cause of worldwide death in industrialized countries. The overall mortality related to myocardial infarction (MI) has largely decreased over the years due to effective procedural therapy and secondary prevention. Unfortunately, for the patients that survive after MI, the morbidity leading to heart failure (HF) is rising leading to increased cost burden on the healthcare system. Moreover, according to the World Health Organization, this burden will be worsened since CVD are predicted to stay at the first rank until 2030 because of an aging population and an exponential growing number of diabetic patients^1,2^.

Acute MI occurs as a consequence of the abrupt occlusion of a coronary artery leading to ischemic injury and subsequent necrosis. Resulting infarct size is correlated to the duration of ischemia and is a determinant of post-MI mortality. Limitation of infarct size by reperfusion of the culprit artery as early as possible (thrombolysis or primary angioplasty) is the only treatment providing myocardial salvage and improving patient prognosis^3,4^. However, reperfusion therapy leads to deleterious side effects, namely ischemia–reperfusion (IR) injury, adding to ichemic injury and limiting myocardial salvage^5^. Upon the abrupt restoration of blood flow, cardiac cells endangered by ischemia die after activation of intracellular cascades of complex events such as calcium overload, the loss of ion homeostasis and the production of oxygen free radicals leading to apoptotic processes. Importantly, lethal IR injury arise when apoptosis is triggered by reperfusion in cells that have survived ischemia^6,7^. Because infarct expansion due to IR injury concerns 25 to 50% of the total infarct, new cardioprotective strategies applicable at the onset of reperfusion are urgently needed.

Thus, targeting an apoptotic cascade activated at the time of reperfusion appeared as a promising and innovative therapeutic strategy to prevent IR injury and limit infarct size^8–12^. Until now, all clinical trials targeting the mitochondrial intrinsic apoptotic pathway involved in IR injury have not been successful. Even if inhibition of the mitochondrial permeability transition pore (mPTP) opening using Cyclosporin A (CsA) provided encouraging results in many animal models and also in a proof-of-concept study in patients^36–38^, large-scale clinical trials provided negative results showing neither long-term protection nor decreased mortality or prevention of early multiple organ failure (CIRCUS, CYRUS)^39,40^.

Therefore, we have capitalized on the pro-apoptotic key role of the FAS death receptor in the extrinsic pathway since elevated Fas Ligand in the blood of AMI patients activates FAS receptor^6^. In this context, the *death domain associated protein* (DAXX) has been identified as a key element involved in IR injury in many organs including the heart^13–15^. The DAXX protein has a dual function depending of its subcellular localization. DAXX in the nucleus acts as a transcription regulator with a main anti-apoptotic contribution^16^. However, triggered by various stimuli such as oxidative or ischemic stresses, DAXX is relocalized into the cytosol upon the *Apoptosis signal-regulating kinase 1* (ASK1)-shuttling^17–21^. Cytosolic pro-apoptotic DAXX protein binds to the intracellular part of the *First Apoptosis Signal* (FAS) death-receptor and activates the downstream apoptotic signaling pathway^22^.

In a previous study, we have identified FAS:DAXX interaction as a key player during myocardial IR injury using the DAXX-dominant negative mice model exhibiting a drastic decrease in infarct size (45%)^13^. In order to translate this genetical into a pharmacological approach, we have developed the anti-apoptotic peptide Tat-DAXXp (TD) composed of the association of a FAS:DAXX interfering peptide DAXXp with the Tat cell-penetrating peptide. After a single intraveneous administration prior to reperfusion, TD-treatment provided a 48%-decrease in infarct size and an inhibition of apoptosis by the same extent in a short-term murine myocardial IR in vivo model compared to a non-treated control group^23^.

Fluorescent labeled TD peptide shows a cytoplasmatic localization in cardiomyocytes in vitro and in vivo. Within the cell, TD peptide ensures cardioprotection by inhibiting both extrinsic and intrinsic apoptotic cascades, reducing JNK, Caspases 8, 9 and 3 activation, and decreasing FADD and BAD expression. TD-induced caspase 8 inhibition was not associated with any switch to necroptosis^23^ (no activation of RIP1, RIP3 or pMLKL) compared to the non-treated IR condition. In addition, this treatment allows activating pro-survival proteins (ERK and AKT) from the RISK pathways. Finally, TD treatment was associated with DAXX re-localization from the cytosol to the nucleus (where it is reported to play anti-apoptotic functions) probably as a consequence of the inhibition of HSP70-mediated proteotoxic stress. This suggests that TD plays a key role in early cardioprotection by strongly inhibiting apoptosis specifically induced by IR injury. Importantly, 24 h after TD administration, the peptide is completely degraded in vitro and eliminated in vivo avoiding possible side effects. Therefore, anti-apoptotic TD peptide is fully active and necessary only at the onset of reperfusion within a 30 min-therapeutic time window, corresponding to the apoptotic burst in the subacute phase of AMI^23^.

The aim of our present study was to evaluate the long-term cardioprotection provided by one bolus injection of TD in mice submitted to 40-min myocardial infarction followed by 6-month reperfusion. In order to optimize the translation into the clinical setting^24^, a moderate IR murine model was used, as a more relevant study model to mimic the majority of AMI patients benefiting from culprit coronary artery revascularization. Moreover, mild injury in the cardiac tissue is compatible with a therapeutic reversion^24^. TD was administrated 5 min before reperfusion (bolus of 1 mg/kg) compared to two placebos, a pharmacological and a surgical one (three-arms study) in a double-blind manner as usual in a clinical trial. Cardiac injury was evaluated by measuring the plasmatic cardiac troponin I (cTnI) biomarker and cardiac function by echocardiography during 6 months after MI. Finally, fibrosis was quantified in the left ventricle (LV) of the mice by histological coloration.

## Results

### Study design

**Figure 1:**
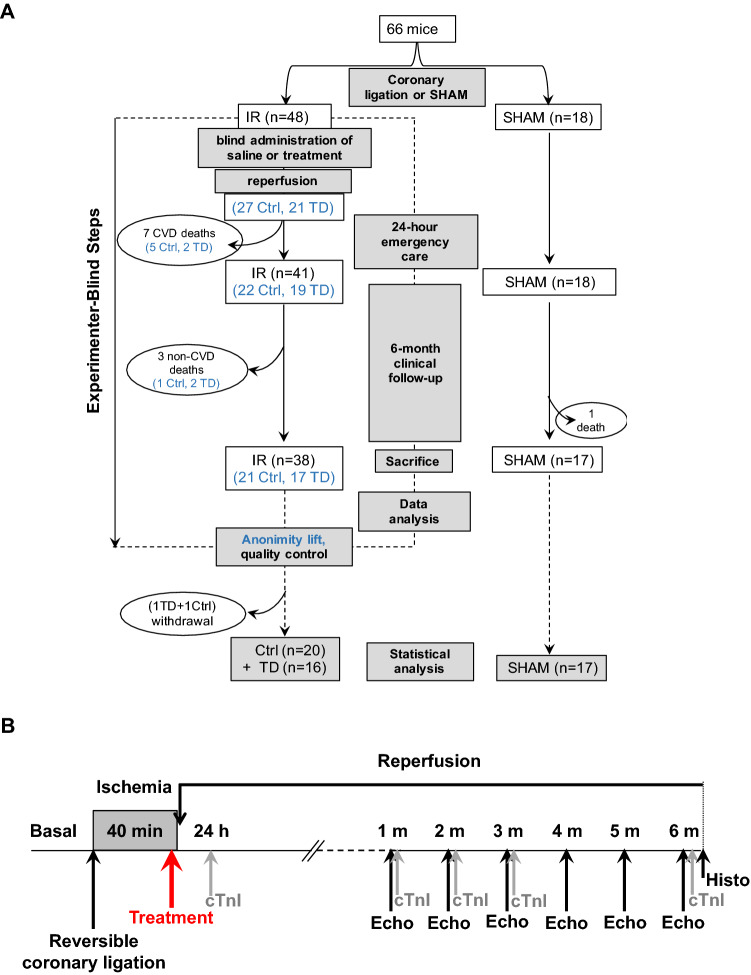
Study design and experimental protocol. (**A**): Study design. Mice (n = 66) were included into the SHAM (surgery placebo; n = 18) or the IR (ischemia–reperfusion, n = 48) sub-groups. In the IR group (with coronary artery ligation), injection of saline buffer (Ctrl) or TD peptide (TD) was blinded to the experimenter by assigning an anonymity number to each mouse. Anonymity was lifted after the completion of all steps of follow-up of the IR group (dotted frame) including data analysis, just before the statistical tests. At this step only, each mouse included in the study was assigned to a study group and the “n” number of mice in each group mentioned in blue was determined. Two mice had to be removed from the study (1 TD and 1 Ctrl) at this step since the quality control applied to the body temperature recordings revealed a slight decrease during myocardial infarction (MI). (**B**): Experimental and treatment protocols. MI was induced by transient coronary artery ligation and reperfusion. Peptide treatment (red arrow) was administered intravenously 5 min before the onset of reperfusion. Blood samples were collected at 24 h, 1, 2, 3 and 6 months post-surgery to assess cardiac Troponin I (cTnI) level. Echocardiographic recordings were performed at 1, 2, 3, 4, 5 and a 6 months post-surgery. At the end of the protocol, a histological analysis was performed. For SHAM animals, the protocol was the same except that there was no coronary ligation and no treatment.

Our study was designed to evaluate the long-term effects of the TD peptide in mice subjected to mild myocardial IR. The studied mice population included the SHAM (n = 18) and the IR groups (n = 49) (Fig. 1A). In the IR group, mice received 5 min before reperfusion either a bolus of saline (non-treated control group, noted Ctrl) or a bolus of TD peptide (1 mg/kg; TD group) in a blinded manner to eliminate experimental biases. After surgery, mice were allowed to recover in an emergency care cabinet and entered in the clinical follow-up study during 6 months (Fig. 1B). All parameters during the pre-clinical follow-up were measured in the same manner in the SHAM, Ctrl and TD mice.

### Decreased cardiac injury

**Figure 2:**
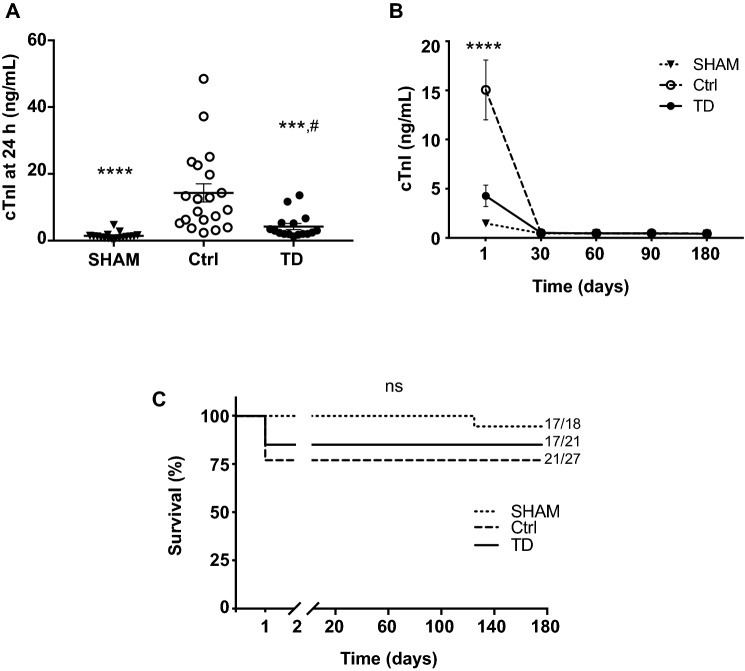
Decreased cTnI levels and evaluation of cohort survival. (**A**): Cardiac Troponin I (cTnI) levels were measured using the high sensitivity mouse cardiac Troponin-I Elisa on plasmatic fractions of blood samples from SHAM (n = 17), Ctrl (n = 20) and TD (n = 16) mice. Scatter dot blots and mean ± SEM were plotted for cTnI measured at 24 h (peak level). Statistical analysis was performed using ANOVA test with the Tukey’s post hoc test for multiple comparisons at 24 h. *** was noted for *p* = 0.0009 and **** for *p* < 0.0001 for TD versus Ctrl and # for *p* = 0.5527 for TD versus SHAM. (**B**): Mean values of cTnI levels evaluated at 1, 30, 60, 90 and 180 days during the clinical follow-up were plotted in SHAM (n = 13), Ctrl (n = 17) and TD (n = 13) mice. Statistical analysis was performed using Two-way ANOVA followed by the Tukey’s post hoc test. **** is given for *p*_group_ < 0.0001. (**C**): Survival curves are presented for SHAM, Ctrl and TD mice. Numbers represent total number of animals that survived (left) relative to those that were included in the study (right). The comparison between the 3 survival curves was performed using the Gehan–Breslow–Wilcoxon test. ns was noted for *p* = 0.2452.

Cardiac troponin I (cTnI) is a well-established biomarker for infarct size with reliable prognostic value both in the clinical setting and in small animals^25–27^. TD-treatment reduced by 70% the amount of cTnI level measured at 24 h when compared to the untreated control (Ctrl) group (4.24 ± 0.90 ng/mL, n = 16 for TD vs. 14.31 ± 2.72 ng/mL, n = 20 for Ctrl; p*** = 0.0009; Fig. 2A) and reached a mean value similar to the SHAM group (1.41 ± 0.24 ng/mL, n = 17 for SHAM vs. TD*; p*^*#*^ = 0.5527). At 1, 2, 3 and 6 months, cTnI levels were close to zero in all study groups (Fig. 2B). During the 6-month follow-up period, survival was not statistically different among groups (*p* = 0.2452; Fig. 2C) despite the 49%-decrease in mortality observed during the first 24 h post-MI period: 9.5% (2/21) for TD versus 18.5% (5/27) for the Ctrl group. Indeed, after the critical period of 24 h post-surgery, all other animals survived during the 6-month post-surgery.

**Figure 3:**
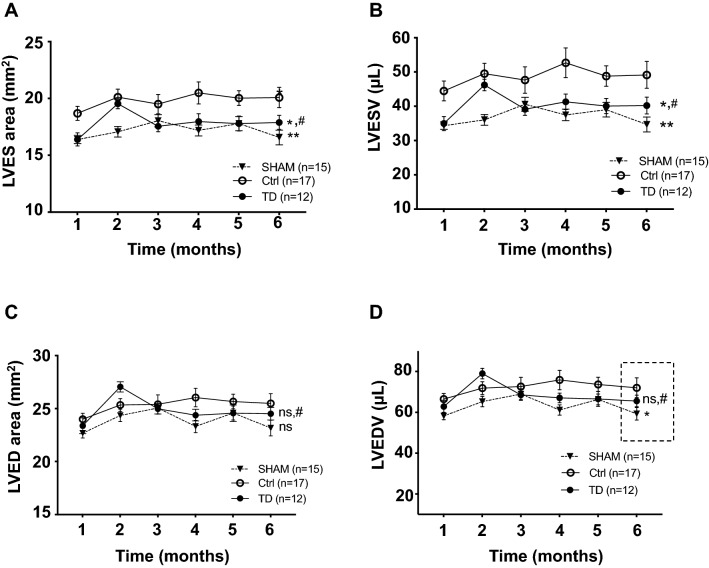
Decreased cardiac dilatation upon TD-treatment. Measurements performed by conventional echocardiography on bi-dimensional images of the parasternal long axis (B-mode) using Vevolab software for LV end-systolic (**A**,**B**) and end-diastolic (**C**,**D**) cavity dimensions in SHAM (n = 15), Ctrl (n = 17) and TD (n = 12) mice. Graphs showing longitudinal changes for (**A**): LV end-systolic area (LVES area, mm^3^) with p** = 0.0012 for SHAM versus Ctrl, p* = 0.0280 for TD versus Ctrl and *p*^#^ = 0.6366 versus SHAM; (**B**): for LV end-systolic volume (LVESV, µL) with p** = 0.0012 for SHAM versus Ctrl, p* = 0.0337 for TD versus Ctrl and *p*^#^ = 0.5868 versus SHAM; (**C**): for LV end-diastolic area (LVED area, mm^3^) with *p*^ns^ = 0.0874 for SHAM versus Ctrl, *p*^ns^ = 0.7571 for TD versus Ctrl and *p*^#^ = 0.4055 versus SHAM; (**D**): for LV end-diastolic volume (LVEDV, µL) with p* = 0.0364 for SHAM versus Ctrl, *p*^ns^ = 0.5560 for TD versus Ctrl and *p*^#^ = 0.3835 versus SHAM. Data are presented as mean ± SEM and connecting lines. Statistical analysis for (**A**,**B**,**C**) was performed using the Two-way ANOVA test followed by the Tukey’s post test for repeated measures. Statistical analysis for (**D**; LVEDV with p_interaction_* = 0.0282) was obtained using one-way ANOVA at 6 months.

**Figure 4:**
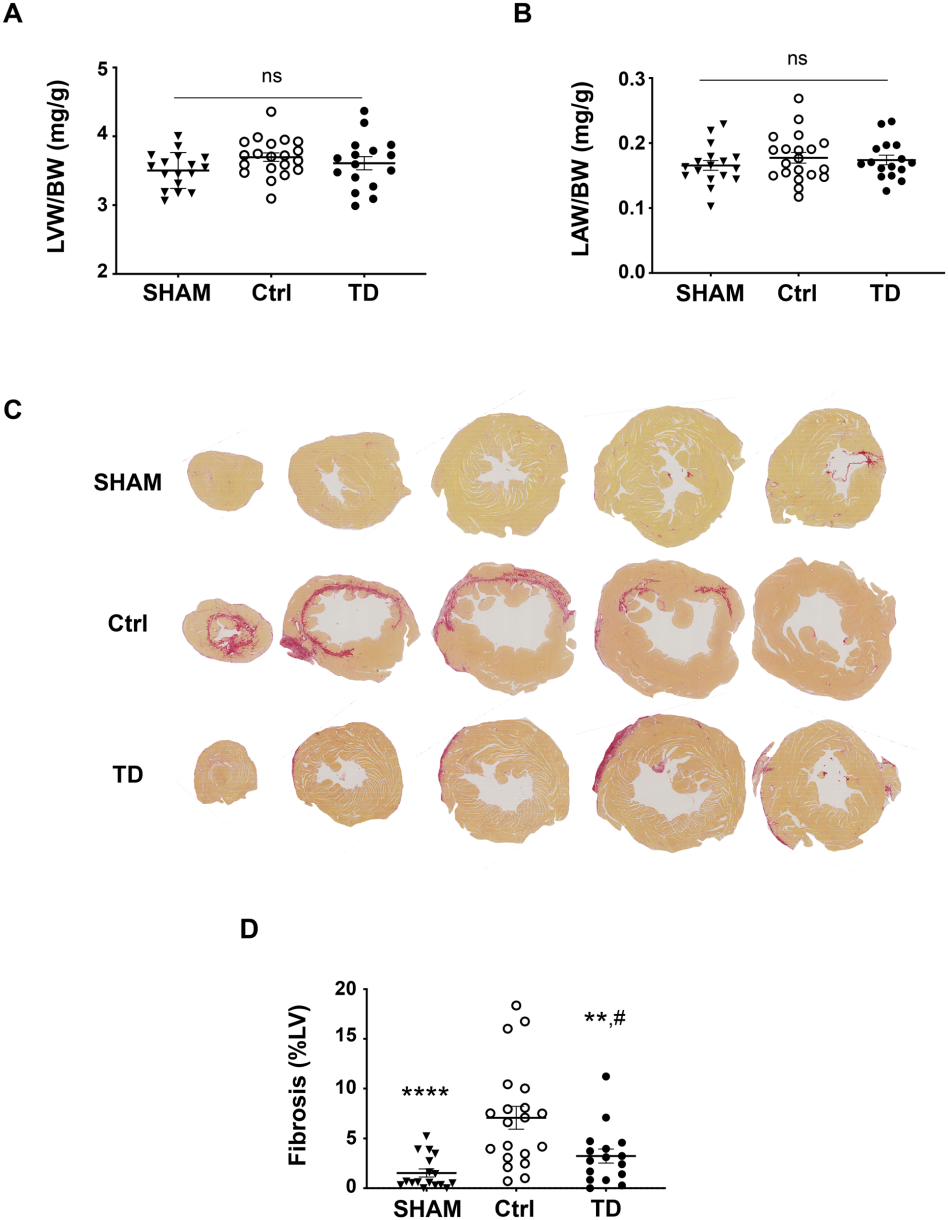
Decreased heart fibrosis in TD-treated mice. At the end of the study, hearts were harvested in SHAM (n = 17), Ctrl (n = 20) and TD (n = 16) mice and weighted. Scatter dot blots and mean ± SEM were plotted for LV and left atria (LA) weights reported to body weight in (**A**): LVW/BW with *p*^ns^ = 0.1707 and in (**B**): LAW/BW with *p*^ns^ = 0.5561. (**C**): Histology was performed on paraffin-embedded sections from LV harvested at 6 months post-MI. Representative pictures of microscopic observations for Picrosirius red stained sections from SHAM, Ctrl and TD hearts. (**D**): Fibrosis area (Scatter dot blots and mean ± SEM) was decreased by 54% in TD-treated versus Ctrl LV samples. Statistical analysis was performed using one-way ANOVA with p**** < 0.0001 for SHAM versus Ctrl, p** = 0.0079 for TD versus Ctrl and *p*^#^ = 0.3795 versus SHAM.

Since structural changes occur in the left ventricular (LV) myocardium after the IR insult in patients, the LV anatomy was evaluated by echocardiography during the post-MI period. Significant LV changes were observed among groups as assessed for both end-systolic area (LVES area; *p*_group_** = 0.0012) and end-systolic volume (LVESV; *p*_group_** = 0.0012) measurements. Indeed, 10% and 17% decreased mean values were observed in the TD-treated mice compared to Ctrl, respectively, for LVES area (TD, n = 12 vs. Ctrl, n = 17, p* = 0.0280) and for LVESV (TD, n = 12 vs. Ctrl, n = 17, p* = 0.0337 allowing to recover mean values close to those of SHAM (TD, n = 12 vs. SHAM, n = 15 with *p*^#^ = 0.6366 for LVES area and *p*^#^ = 0.5868 for LVESV; Fig. 3A,B). There was no significant difference between TD and Ctrl groups for LV end-diastolic area (LVED area: *p*^ns^ = 0.7571; Fig. 3C) during the 6 month-period. By contrast, LV end-diastolic volume was statistically different among groups during the follow-up (LVEDV: *p*_group_* = 0.0467 and *p*_interaction_* = 0.0282). At 6 months, LVEDV was increased by 18% in the Ctrl group *versus* SHAM (p* = 0.0317). TD treatment allowed a 9%-decrease for this parameter versus Ctrl to recover mean values close to those of SHAM (*p*^#^ = 0.7753) without reaching significance (*p*^ns^ = 0.1558, TD vs. Ctrl; Fig. 3D). Regarding the mass of cardiac chambers, there was no difference among groups at 6 months for left ventricular weight/body weight (LVW/BW; *p*^ns^ = 0.1707; Fig. 4A) and left atria weight/body weight (LAW/BW; *p*^ns^ = 0.5561; Fig. 4B). Same results were observed for the measurements of cross-sectional area evaluated on hematoxylin/eosin-stained LV sections showing the absence of hypertrophy in the left ventricles after IR injury (Figure S1). Histopathological changes induced by IR injury were also investigated by evaluating fibrosis formation on LV slices after Picrosirius red staining at 6 months post-reperfusion (Fig. 4C). In the LV of mice treated with a bolus of TD (1 mg/kg) 5 min prior to reperfusion, the percentage of fibrosis was evaluated and compared between groups (*p*_group_**** < 0.0001). Importantly, TD treatment induced a large 54% reduction in fibrosis formation compared to non-treated Ctrl hearts (p** = 0.0079 for TD, n = 16 vs. Ctrl, n = 20; Fig. 4D). The amount of fibrosis in TD-treated mice corresponded nearly to the SHAM condition (*p*^#^ = 0.3795 for TD, n = 16 vs. SHAM, n = 17).

### Improved post-MI functional recovery

**Figure 5:**
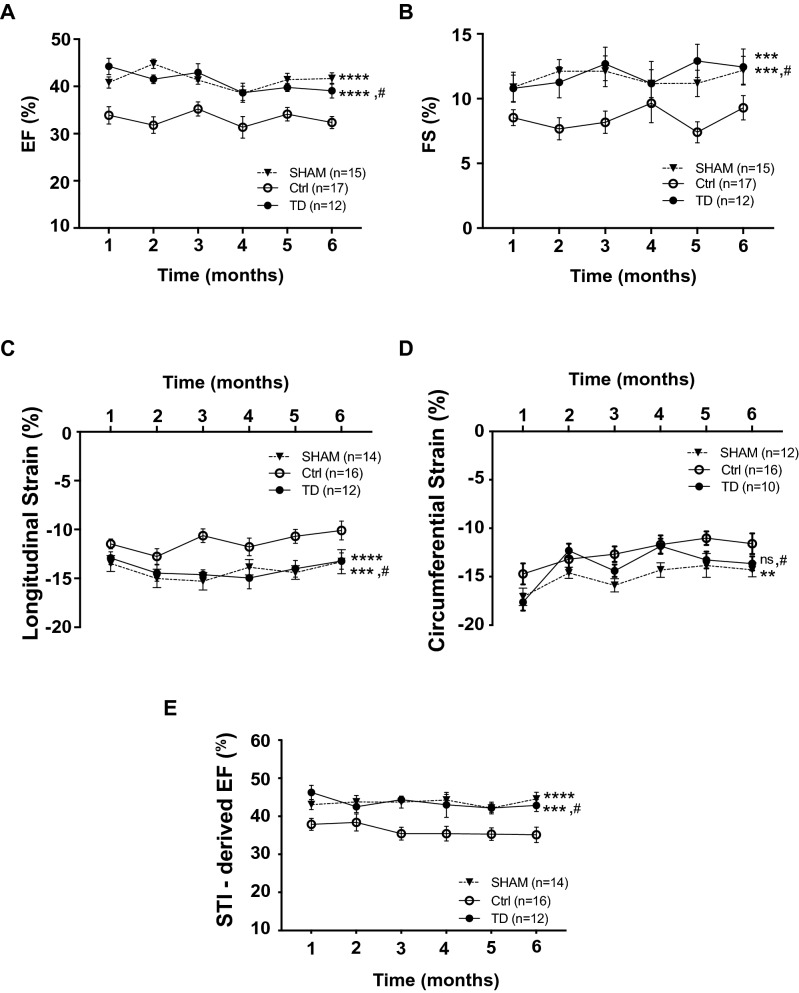
Improved cardiac performance in TD-treated mice. Echocardiographic parameters were calculated from end-diastolic and end-systolic LV volumes using Vevolab software in SHAM (n = 15), Ctrl (n = 17) and TD (n = 12) mice. (**A**): ejection fraction (EF, %) with p**** < 0.0001 for SHAM versus Ctrl, p**** < 0.0001 for TD versus Ctrl and *p*^#^ = 0.9621 versus SHAM and (**B**): fractional shortening (FS, %) with p*** = 0.0005 for SHAM versus Ctrl, p*** = 0.0004 for TD versus Ctrl and p^#^ = 0.9466 versus SHAM. Speckle-tracking imaging using Vevostrain software allowed to evaluate (**C**): the global longitudinal strain (peak average, in %) from a parasternal long axis view in SHAM (n = 14), Ctrl (n = 16) and TD (n = 12) mice with p**** < 0.0001 for SHAM versus Ctrl, p*** = 0.0003 for TD versus Ctrl and *p*^#^ = 0.9562 versus SHAM; (**D**): the global circumferential strain (%) from a parasternal short axis view in SHAM (n = 12), Ctrl (n = 16) and TD (n = 10) mice with p** = 0.0096 for SHAM versus Ctrl, *p*^ns^ = 0.2557 for TD versus Ctrl and *p*^#^ = 0.4163 versus SHAM. STI allowed also to record an automated measurement of ejection fraction (STI-derived EF, %) obtained from endocardial detection in end-diastole and end-systole in SHAM (n = 14), Ctrl (n = 16) and TD (n = 12) mice in (**E**) with p**** < 0.0001 for SHAM versus Ctrl, p*** = 0.0002 for TD versus Ctrl and *p*^#^ = 0.9987 versus SHAM. Statistical significance was tested using Two-way ANOVA followed by the Tukey’s post test for repeated measures. Data are presented as mean ± SEM and connecting lines.

Histopathological and structural changes occurring in the myocardium after IR insult lead to progressive decline in LV performance. Ejection fraction (EF, %) and fractional shortening (FS, %), which are considered as reliable assessments of LV function^28,29^, were calculated from both conventional and strain parameters using the bi-dimensional mode. We observed significant changes among groups (*p*_group_**** < 0.0001) for EF which is the main parameter reflecting systolic function (Fig. 5A). Indeed, EF was significantly increased by 24% in the TD group versus Ctrl along the 6 month-period (p**** < 0.0001 for TD, n = 12 vs. Ctrl, n = 17) and more importantly, mean values were close to those recorded in the SHAM group (*p*^#^ = 0.9621 for TD, n = 12 vs. SHAM, n = 15). A similar result was observed for FS (*p*_group_**** < 0.0001) exhibiting a drastic 40%-increase upon TD treatment compared to non-treated Ctrl mice (p*** = 0.0004 for TD, n = 12 vs. Ctrl, n = 17 and *p*^#^ = 0.9466 vs. SHAM, n = 15; Fig. 5B).

In mice recovering from MI, strain-based echocardiography is considered as more sensitive than conventional echocardiography to assess cardiac function and efficacy of cardiac therapy^30^. In our study, TD treatment significantly improved strain properties that were strongly impaired after IR injury (*p*_group_**** < 0.0001 for both longitudinal strain and STI-derived EF; *p*_group_* = 0.0126 for circumferential strain). Indeed, a 25%-increase in the longitudinal strain average (on a parasternal long axis view) was observed in the TD group compared to Ctrl (p*** = 0.0003 for TD, n = 12 vs. Ctrl, n = 16 and *p*^#^ = 0.9562 vs. SHAM, n = 14; Fig. 5C).

Circumferential strain, which represents the percent change in myocardial circumference measured from a parasternal short axis view, was also decreased by IR-injury (17%; p** = 0.0096 for SHAM, n = 14 vs. Ctrl, n = 16) but TD treatment was not able to fully restore this strain property (11%; *p*^ns^ = 0.2557 for TD, n = 10 vs. Ctrl, n = 16; Fig. 5D). Regarding strain evaluation on the radial axis, there was no difference among groups in our study (*p*_*group*_^ns^ = 0.1768; Supplemental Figure S2). Global LV function was also assessed from averaging of segmental 2D-strain from endocardial detection in end-diastole and end-systole of speckle tracking (STI-derived EF). Results show again a clear improvement of global LV function (20%-increase) upon TD treatment compared to Ctrl (p**** < 0.0001 for TD, n = 12 vs. Ctrl, n = 16 and *p*^#^ = 0.9987 vs. SHAM, n = 14; Fig. 5E).

## Discussion

Our study evaluated, for the first time through a 6-month follow-up in a mouse model of myocardial ischemia–reperfusion (IR) injury, the long-term cardioprotective effects of the anti-apoptotic Tat-DAXXp (TD) peptide injected as an intravenous bolus (1 mg/kg body weight). The results of this study performed in a double-blind manner demonstrate that TD-treatment enhanced the beneficial effect of reperfusion up to 6 months following injection in a relatively mild model of IR injury. TD-treatment applied as a bolus, 5 min before reperfusion, reduced both cardiac injury (cTnI level) and remodeling (fibrosis). Accordingly, left ventricle (LV) global function of the myocardium was significantly improved along the 6-month period as showed by a significant higher ejection fraction (EF, %). These new data further expand and provide relevance for clinical translation to our previous results showing short-term cardioprotective effects associated to reduced mortality rates measured at 24 h post-MI^31^.

The TD peptide was designed to specifically act on the FAS:DAXX apoptotic pathway that is activated specifically during myocardial reperfusion injury. In mechanistic terms, our previous study evidenced that short-term cardioprotection was related to a potent anti-apoptotic effect by targeting the FAS receptor-downstream pathway (decreased activation of caspases 8 and 3) associated with an activation of RISK survival kinases^31^. In addition, due to a partial inhibition of caspase 8, we did not observe a switch to necroptosis that could bypass the effect of the treatment (no changes in RIP1, RIP3 and pMLKL protein level). Furthermore, inhibition of the mitochondrial apoptotic pathway was also evidenced (decreased activation of caspase 9) due to the crosstalk between the two main apoptotic cascades. This therapeutic strategy allows an upstream inhibition of regulated cell death mechanisms.

Decreasing infarct size in patients allows the prevention of post-ischemic heart failure (HF) and the improvement of survival. LV remodeling is dominated during the myocardial infarction (MI) acute phase by an early dilatation of the ventricle (a key marker of the evolution and prognosis of the disease) followed by deposit of scar fibrosis to replace the injured tissue and hypertrophy in non-infarcted remote areas. Therapeutic strategies for MI patients must be implemented as early as possible to avoid ventricular dilatation. As described in the literature, the duration and extent of MI must be reduced by rapid heart revascularization, and IR injury resulting from both apoptosis and inflammation must be treated^31–33^. Results obtained in the present study confirm that decreasing IR injury, specifically when reperfusion starts, provides long term protection of the myocardium by decreasing cardiac injury in the early post-MI period and preventing subsequent heart remodeling.

In patients, cardiac specific troponin I (cTnI) is considered as a diagnostic and prognostic biomarker for cardiac pathology as well as an essential indicator of the risk stratification of acute MI^25,26^. cTnI dosage in patients with acute coronary syndrome allows the early recognition of those with an increased risk of death^34^. In murine models, cTnI is also a prognostic value for infarct size^27^. Determination of plasma levels at 24 h was reported to be an early predictor of infarct size in mice with either permanent or transient ligation of the left ascending coronary artery^35,36^ and described as a biomarker of cardiotoxicity in preclinical models^37^. In the present study, cTnI plasma levels was decreased to 70% in TD-treated IR mice (TD group) compared to the Ctrl (non-treated IR) group (Fig. 2A) at 24 h, in agreement with the reduced infarct size and post-MI cardiac mortality reported in our previous short-term study^31^. From 1 to 6 months, cTnI levels were close to zero (Fig. 2B) in all study groups confirming the absence of cardiac injury after MI and TD-treatment.

Following myocardial infarction leading to the death of cardiac cells, macrophages remove necrotic tissue, which is then replaced by granular tissue to provide a fibrotic scar. In our mouse model of ischemia–reperfusion injury, the amount of fibrosis was increased as evidenced in the Ctrl group. We did not observe any impact of the deposit of fibrosis on the diastolic stiffness of the LV as attested by our echocardiography study using the pulse-waved Doppler mode clearly showing that the mitral valve E/A ratio considered as a clinical marker of diastolic dysfunction was similar upon IR stress. Our results gave evidence that the enhanced fibrosis in Ctrl versus SHAM left ventricles upon myocardial injury, as investigated *post mortem* at the end of the protocol, was drastically decreased in the TD-treated hearts. Myocardial fibrosis is a major parameter of remodeling after MI^38^. During this process, injured myocardial wall becomes thinner and the left ventricle dilates. The non-infarcted myocardium is also altered by the deposit of interstitial fibrosis together with cardiomyocyte hypertrophy. These structural alterations lead to cardiac dysfunction, which could progress to heart failure (HF).

As a direct result of systolic dysfunction related to cardiac remodeling, end-systolic left ventricle enlarges and LVESV (LV end-systolic volume) changes are considered as prognostic values in cardiac remodeling^39,40^. In our study, echocardiographic evaluation of end-systolic area (LVES area) and LVESV during the 6-month period after MI revealed the expected LV dilatation in the non-treated Ctrl group compared to SHAM, which was prevented by TD-treatment from the first month after MI (Fig. 3). A the end of the follow-up, our data show that LVEDV was increased by 18% in the Ctrl group *versus* SHAM suggesting mild cardiac remodeling in the non-treated IR group. This value is close to the threshold value (20%) used in clinical practice for LV remodeling^41^. There was a trendancy for TD treatment to decrease LVEDV mean value at 6 months, which allows to prevent post-MI LV remodeling. A bad prognosis for patients is correlated with both increased end-systolic and end-diastolic volumes but LVESV is considered as a stronger predictor of adverse outcomes and the primary predictor of survival after MI^42^. In our study, we have not observed any differences in the survival rates during the 6 month-period, in particular by comparing the Ctrl and the SHAM groups, since we have choosen a mild injury model in accordance with the recommendations provided to translate cardioprotection into the clinical setting. Nowadays, it has been widely discussed that obtaining positive results in clinical trials depends on the recruitment of patients who have truly a chance to benefit from adjunct cardioprotection^24^. Indeed, patients with severe injury (with long ischemic period) has less chances to benefit of an effective therapeutic-based cardioprotection that can be additive to reperfusion treatment. In addition, severe injury is associated with strong necrosis induction that could not be reversed by any cardioprotective therapeutic strategy. According to this idea, we have adapted our study mouse model by inducing myocardial IR injury without progression to heart failure and used a functional investigation technique as in humans (Strain echocardiography to detect subtle changes) in order to facilitate the translation from bench to bedside. In addition, compared to permanent LAD ligation, our IR model is more clinically relevant because most of the AMI patients nowadays benefit from revascularization of their culprit coronary artery. In particular, criteria for patient selection should be short ischemic stress (< 2–3 h), large area-at-risk (> 30–40% of LV) and no significant collaterality, which corresponds to our mouse IR model^24^. In addition, no hypertrophy was detected in the hearts and no mouse died of cardiovascular cause beyond the immediate post-infarction critical period (first 24 h of recovery). In accordance, absence of significant changes in LVED volume and area (conventional echocardiography) were observed in Ctrl mice from 1 to 6 months compared to SHAM mice. These results show that the study model of 40 min myocardial ischemia followed by 6-month reperfusion is characterized by the absence of bad prognosis, which is usually reported in mouse models of permanent LAD (Left Anterior Descending) artery ligation leading to heart failure and high mortality rates.

Literature describes that cardiac remodeling, which is correlated to infarct size, impairs cardiac function after MI^43^. As expected, the early reduction in cardiac injury reflected by reduced cTnI plasmatic levels (marker of infarct size) after TD-treatment observed in our study was followed by a marked improvement in LV systolic performance. The ejection fraction (EF, %) parameter is considered as the main marker of cardiac performance and is widely used to easily assess global LV function. In addition, EF evaluation allows to predict mortality and plays a critical role in clinical decisions including device implantation and valve surgery^44^. In the present study, EF was measured from the bi-dimensional mode by two different methods using the LV trace module and the area-length method^45^. A higher EF mean value was observed in TD-treated mice at each time point compared to those of Ctrl mice showing a clear treatment-related long-term improvement of cardiac performance after MI (Fig. 5A).

Strain measurements evaluate the changes in deformation over the cardiac cycle and detect regional cardiac abnormalities occurring even if chamber dimensions, wall thickness or ejection fraction are unchanged^30,46,47^. Moreover, speckle tracking echocardiography provides a more sensitive assessment of cardiac integrity and function post-MI in non-treated and treated mice versus conventional echocardiography^30^. Strain echocardiography evaluates the deformation of the myocardium from continuous frame-by-frame tracking of acoustic speckles (speckle tracking imaging or STI) using the two-dimensional strain (2D-strain) method, which is angle-independent and less subject to artefacts^48^. Strain analysis evaluates cardiac performance, which depends on three factors: ventricular geometry, myocardial fiber orientation (longitudinal in the sub-endocardium, radial in the sub-epicardium and circumferential in the middle of the wall) and wall elasticity^28^. In patients with previous MI, strain imaging allows prediction of clinical outcome better than LVEF parameter^49^. In particular, decreased longitudinal strain predicts mortality in high-risk MI patients^50^. In mice, a previous study reported that longitudinal strain was correlated with infarct size and that this correlation was greater than for radial strain^51^. In addition, longitudinal strain was also described as a highly sensitive parameter to early detect myocardial dysfunction after MI as well as beneficial cardioprotective effects^30^. Thus, we used strain echocardiography for a more detailed investigation of myocardial function. We confirmed that in our model of coronary occlusion longitudinal strain values were more significantly impacted (p****) than those for circumferential strain (p*) versus SHAM in agreement with the higher sensitivity to ischemia of the longitudinal fibers located in the endocardial and subendocardial tissues than the circumferential ones. In addition, we did not detect significant changes among groups for the radial strain parameter as previously described^50,51^. Interestingly, our results show an improvement of 2D longitudinal strain in TD-treated versus non-treated Ctrl mice (Fig. 5C).

Altogether, our data evaluating both conventional and strain parameters clearly indicate that TD-treatment improved global LV function compared to the non-treated (Ctrl) mice. All the results for plasma cTnI levels, fibrosis amount and functional echocardiography show that a single dose of the anti-apoptotic TD peptide administered systemically prior to reperfusion constitutes an effective treatment against LV remodeling after myocardial infarction with a persistence of the cardioprotective effect over a 6-month period.

In conclusion, a single dose of TD peptide prior to reperfusion decreases infarct size assessed by cTnI quantification and induces a restoration of cardiac performance together with a prevention of post-MI LV remodeling. Importantly, we did not detect behavorial changes during the 6-months period or abnormalities such as tumor development and organ hypo- or hypertrophy among mice at the end of the study protocol, suggesting the safety of this potential new therapeutic tool. In addition, specifically targeting the burst of apoptosis involving FAS:DAXX interaction at the onset of reperfusion is safe because TD peptide is administrated as a single bolus and it has a short life time as reported in our previous study^31^. Therefore, TD-treatment appears as a promising therapeutic molecule to prevent post-MI reperfusion injury which should be tested in a clinical situation.

## Limitation of the study

Our study was performed using young adult male mice in order to avoid the influence of the cardioprotective female hormons. However, we need to consider that in the clinical setting, women are also a population at risk of cardiovascular disease. In addition, for a future preclinical study, we need also to take into account that animal studies should be performed on old animals with co-morbidities and medications usually prescribed in high cardiovascular risk patients for an optimal clinical translation.

## Methods

All experiments were carried out on C57BL/6 J mice (*Charles River laboratory, l'Abresle, France*) in accordance with the European Communities Council directive of November 1986 and conformed to the “Guide for the Care and Use of Laboratory Animals” published by the US National Institutes of Health (NIH publication 8th Edition, 2011). The study protocols were approved by the “Comité d’éthique pour l’expérimentation animale Languedoc-Roussillon” (CEEA-LR) with the authorization number CEEA-LR-12107 for experiments to be realized on the site of experimentation that obtained authorization number A 34-172-41.

### Surgery

Investigations conform to the Directive 2010/63/EU of the European Parliament. C57Bl6J male mice were subjected to a surgical model of acute myocardial ischemia and reperfusion as previously described^32,52^. Mice (22–30 g, *Charles River, France)* were anaesthetized by an anaesthetic preparation (intramuscular injection) composed by xylazine (10 mg/kg; Rompun 2%; *Bayer, France*), ketamine (50 mg/kg; Imalgène 500; *Merial, France*) and chlorpromazine (1.25 mg/kg; Largactil 5 mg/mL; *Sanofi-Aventis, France*). Mice were placed under mechanical ventilation through a tracheal intubation on a rodent respirator *(Harvard Apparatus, USA*; tidal volume/body mass: 7.2 µL/g; respiratory rate: 200 breaths per min). A thermo-regulated table (connected to a rectal probe) allowed maintaining the body temperature between 36.8 and 37 °C during the surgery. Ketamine (50 mg/kg; Imalgène 500, *Merial, France*) and xylazine (10 mg/kg, Rompun 2%; *Bayer, France*) were injected to fully induce anesthesia before opening the chest by a left lateral thoracotomy and placing a reversible coronary artery snare occluder around the left coronary artery. During the surgical protocol, anesthezia and pain analgesia were controled by pinching the toes of the posterior legs. All mice experienced an ischemic injury of 40 min. The coronary reperfusion was obtained by loosening the silk knot. Then, the chest was sutured after administration of lidocaine (1.5 mg/kg; Xylocaïne 10 mg/mL; *AstraZeneca, France*) subcutaneously. Postoperative awakening was achieved with oxygen supply and controlled humidity in an emergency care unit maintained at 28 °C where mice were allowed to recover during 24 h. Then they were transferred in a ventilated cabinet where they were maintained during all the study.

Various protocols were applied:

- SHAM: sham operation for surgery placebo was performed without coronary artery ligation (surgical placebo).
- Ctrl (non treated IR mice): 40 min ischemia—6 months reperfusion with an I.V. injection of physiological serum 5 min before the onset of reperfusion (pharmacological placebo).
- TD (TD-treated IR mice): 40 min ischemia—6 months reperfusion with an I.V. injection of TD peptide (1 mg/kg) 5 min before the onset of reperfusion.

Mice subjected to the IR protocol were randomly allocated to the two groups of treatment Ctrl or TD (blind to the experimentor).

### Pharmacological treatments

Tat-DAXXp (TD; *Intavis AG, Germany*) was solubilized in physiological serum (NaCl 0.9%; *Lavoisier, France*) to a final concentration of 1 mg/kg. Pharmacological treatments with 15 µL of the peptide solution or physiological serum (NaCl 0.9%; *Lavoisier, France*) were given blindly to the surgery experimentor, who administered them intravenously in the tail vein of the anaesthetized mice 5 min prior to reperfusion.

### Post-surgery clinical follow-up

Mice were subjected to a 6-month follow-up program comprising an echocardiographic evaluation of their functional post-infarction recovery at 1, 2, 3, 4, 5 and 6 months after surgery (Fig. 1B).

#### Blood sampling

200 µL of blood was collected in heparinated tubes from the retro-orbitary sinus of anaesthetized mice (ketamine-xylazine, see above) at various time points (24 h, 1, 2, 3 and 6 months after surgery). After centrifugation at 4 °C, the plasma was collected and stored at -80 °C.

#### Quantification of plasmatic cardiac troponin I

Cardiac troponin I (cTnI) levels were quantified at various time points in the plasma using the *High sensitivity mouse cardiac Troponin-I Elisa immunoassay* kit (*Life Diagnostics, United Kingdom*) and an Infinite M 200 plate reader (*Tecan, France*). The assay was performed in duplicates for each mouse after having collected all samples of the long-term study.

#### Echocardiography

Transthoracic echocardiographic recordings were realized at 1, 2, 3, 4, 5 and 6 months in post-MI mice^53^. Cardiac anatomy and function in vivo were measured non-invasively using a high-frequency, high-resolution echocardiographic system consisting of a VEVO ultrasound machine (1100) equipped with a 22–55 MHz (MS-550D) bifrequencial transducer (*VisualSonics B.V., The Netherlands*). Anesthetized with 1–1.2% isoflurane (*Axience, France*), mice in supine position were placed on an homeothermic stage (body temperature at 37 °C) equipped with electrocardiographic electrodes allowing heart rate monitoring during image acquisition (Table S1). High-resolution images were recorded in the parasternal long and short axes, apical and supra-sternal orientations. B-mode measurements were based on endocardial outlining on both parasternal long axis (PLAX) and short axis (PSAX) by Vevolab software (LV trace) and Vevostrain software (2D-strain) (*VisualSonics B.V., The Netherlands*).

Based on LV end-systole area (LVES area, mm^2^) and LV end-diastole area (LVED area, mm^2^), end diastolic (LVESV, µL) and end-systolic LV volumes (LVEDV, µL), ejection fraction (EF,%) was calculated. Global longitudinal and radial strains were measured from the bidimensional long axis view of the left ventricle using the *Vevostrain* software and the speckle tracking imaging. Circumferential and radial strains were measured from the bidimensional short axis view and these measured values were used to calculate STI-derived ejection fraction (EF,%) (see Tables S1, S2 for all values).

### Mouse euthanasia

At the end of the complete protocol, mice were weighted and anesthetized by administrating (intramuscular injection) a solution composed of ketamine (50 mg/kg; Imalgène 500, *Merial,* France) and xylazine (10 mg/kg; Rompun 2%, *Bayer, France*) and maintained on a surgical table under ventilation. Euthanasia was performed by harvesting the heart after opening of the chest. After rinsing the heart with a PBS solution, the right ventricle and the atria were removed. Cardiac chambers were weighted and the left ventricle processed for fibrosis quantification (see below).

### Histology

Left ventricles (LV) were fixated in a 4%-PFA solution (Electron Microscopy Sciences) during 48 h and embedded in paraffin before slicing (RHEM platform, Biocampus, Montpellier, France). 3 µm-sections were stained with Picrosirius red (RHEM platform, Biocampus, Montpellier, France). Fibrosis revealed by Picrosirius staining was quantified by measuring the extent of fibrotic area in each LV cross-sections using an ImageJ macro developed by MRI platform (Biocampus, Montpellier, France). For each slice, total fibrosis area was corrected to the total LV area. The amount of total fibrosis in each LV was obtained by averaging the percentage of fibrosis of all the slices across the whole LV. Finally, fibrosis values from Ctrl and TD LVs were corrected by subtracting the value measured in the SHAM group corresponding to the perimeter fibrosis. This allows to exclude the unspecific fibrosis of adherence resulting from post-surgery healing of the heart without pericardium in the chest.

Hematoxylin & eosin staining was performed on 3 µm-sections (at the papillary muscle level) and mean cardiomyocyte cross-sectional area was measured for 180 random cells in the non-infarcted region of each LV using Fiji software (*Fiji is just ImageJ*, version 2.1.0/1.53c; https://imagej.net/Fiji).

A whole slide scanner (Nanozoomer 2.0-HT, Hamamatsu) allowed scanning of the stained slides using a × 20 magnification for Picrosirius red and × 40 for HE staining. All the measurements were performed in a blind-manner.

### Statistical analysis

Data (mean ± SEM) were analyzed statistically using GraphPad Prism software (version 8.3.0 for macOs, GraphPad Software, San Diego, California USA, www.graphpad.com).

For parameters evaluated at a single time point, one-way ANOVA, non-parametric Kruskal–Wallis or Unpaired T-test were used to compare the data when appropriate. For parameters measured at multiple time points, statistical analysis was performed using the two-way ANOVA test (parameters: time effect and group effect) in order to evaluate sources of variation among groups. *P* values were given for *p*_*group*_ and multiple comparisons were performed using the Tukey’s post hoc only when *p*_*interaction*_ was non-significant. For survival curve analysis, comparison was performed using the Gehan-Breslow-Wilcoxon test. Statistical significance was noted as ns for *p* > 0.05,* for *p* < 0.05, ** for *p* < 0.01, *** for *p* < 0.001 and **** for *p* < 0.0001.

## Supplementary information

Supplementary Information.

## Data Availability

Materials, data and associated protocols are available to readers.
