# Supplementary material for: Anti-apoptotic peptide for long term cardioprotection in a mouse model of myocardial ischemia–reperfusion injury
Source: Sci Rep. 2020 Oct 22;10:18116. doi: 10.1038/s41598-020-75154-x (PMC7582178; doi:10.1038/s41598-020-75154-x)
Supplement: Supplementary file 1 — Supplementary Information. [file 41598_2020_75154_MOESM1_ESM.docx]

**SUPPLEMENTARY MATERIAL**

**Anti-apoptotic peptide for long term cardioprotection in a mouse model of myocardial ischemia-reperfusion injury**

Aurélie Covinhes^1,2^, Laura Gallot^1,2^, Christian Barrère^1,2^, Anne Vincent^1,2,^, Catherine Sportouch^1,2,3^, Christophe Piot^1,2,3^, Bernard Lebleu^4^, Joël Nargeot^1,2^, Prisca Boisguérin^5,#^ and Stéphanie Barrère-Lemaire^1,2#*^

^1^, IGF, Université de Montpellier, CNRS, INSERM, Montpellier, France;

^2^, Laboratory of Excellence Ion Channel Science and Therapeutics, Valbonne, France;

^3^, Département de Cardiologie Interventionnelle, Clinique du Millénaire; Montpellier, France;

^4^, LPHI, Université de Montpellier, CNRS, Montpellier, France;

^5^, CRBM, Université de Montpellier, CNRS, Montpellier, France.

^#^, considered as joint last authors

^*^ **Corresponding author:**

**Stéphanie Barrère-Lemaire**

**Institut de Génomique Fonctionnelle**

141, rue de la Cardonille - 34094 Montpellier Cedex 5 -France

Tel: +33 4 34 35 92 46 - Fax: +33 4 67 54 24 32

Email: stephanie.barrere@igf.cnrs.fr

**Supplemental Tables**

**Table S1: Data mean values for conventional parameters evaluated by VevoLAB**

**Table S2: Data mean values for Strain parameters evaluated by VevoStrain**

**Table S3: Data mean values for conventional parameters evaluated by echocardiography using the pulsed-wave Doppler mode.**

**Supplemental Figures:**

**Figure S1:** **HE staining on LV slices at 6 months post-MI and evaluation of** **cross-sectional area of the cardiomyocytes.** (**A)** Representative LV slices stained with hematoxylin & eosin (HE) from SHAM, Ctrl and TD groups. (**B**) Representative images of cross-section of longitudinal fibers from the remote non-infarcted area (interventricular septum) shown for each study group and scatter-dot plot and mean ± SD for quantification of cardiomyocyte cross-sectional area (CSA) measured on 180 random cells/heart, n=7 hearts *per* group. Statistical analysis was performed using the non-parametric test Kruskal-Wallis; p = 0.1175. Bar scale is indicated for each panel.

Figure S2: Speckle-tracking imaging using *Vevostrain* software to evaluate radial strain (average, peak %) from a parasternal long axis view in SHAM (n = 14), Ctrl (n = 16) and TD (n = 12) mice. Statistical significance was tested using Two-way ANOVA followed by the Tukey’s *post-test* for repeated measures. Data are presented as mean ± SEM and connecting lines. ns was noted for *p* = 0.1766 for TD *versus* Ctrl, * for *p* = 0.0372 for SHAM *versus* Ctrl, and # for *p* = 0.7947 for TD *versus* SHAM.
